# TRAIL-R2 promotes skeletal metastasis in a breast cancer xenograft mouse model

**DOI:** 10.18632/oncotarget.3321

**Published:** 2015-03-25

**Authors:** Hendrik Fritsche, Thorsten Heilmann, Robert J. Tower, Charlotte Hauser, Anja von Au, Doaa El-Sheikh, Graeme M. Campbell, Göhkan Alp, Denis Schewe, Sebastian Hübner, Sanjay Tiwari, Daniel Kownatzki, Susann Boretius, Dieter Adam, Walter Jonat, Thomas Becker, Claus C. Glüer, Margot Zöller, Holger Kalthoff, Christian Schem, Anna Trauzold

**Affiliations:** ^1^ Division of Molecular Oncology, Institute for Experimental Cancer Research, CCC-North, University of Kiel, Kiel, Germany; ^2^ Department of Gynecology, University Hospital Schleswig-Holstein, Kiel, Germany; ^3^ Section Biomedical Imaging, Department of Radiology and Neuroradiology, University Hospital Schleswig-Holstein, Kiel, Germany; ^4^ Clinic for General Surgery, Visceral, Thoracic, Transplantation and Pediatric Surgery, University Hospital Schleswig-Holstein, Kiel, Germany; ^5^ Department of Tumor Cell Biology, University Hospital of Surgery, Heidelberg, Germany; ^6^ Department of General Pediatrics, ALL-BFM Study Group, University Hospital Schleswig-Holstein, Kiel, Germany; ^7^ Institute of Immunology, University of Kiel, Kiel, Germany

**Keywords:** TRAIL-R2, breast cancer, CXCR4, bone metastasis, bone homing

## Abstract

Despite improvements in detection, surgical approaches and systemic therapies, breast cancer remains typically incurable once distant metastases occur. High expression of TRAIL-R2 was found to be associated with poor prognostic parameters in breast cancer patients, suggesting an oncogenic function of this receptor. In the present study, we aimed to determine the impact of TRAIL-R2 on breast cancer metastasis. Using an osteotropic variant of MDA-MB-231 breast cancer cells, we examine the effects of TRAIL-R2 knockdown *in vitro* and *in vivo*. Strikingly, in addition to the reduced levels of the proliferation-promoting factor HMGA2 and corresponding inhibition of cell proliferation, knockdown of TRAIL-R2 increased the levels of E-Cadherin and decreased migration. *In vivo*, these cells were strongly impaired in their ability to form bone metastases after intracardiac injection. Evaluating possible underlying mechanisms revealed a strong downregulation of CXCR4, the receptor for the chemokine SDF-1 important for homing of cancers cells to the bone. In accordance, cell migration towards SDF-1 was significantly impaired by TRAIL-R2 knockdown. Conversely, overexpression of TRAIL-R2 upregulated CXCR4 levels and enhanced SDF-1-directed migration. We therefore postulate that inhibition of TRAIL-R2 expression could represent a promising therapeutic strategy leading to an effective impairment of breast cancer cell capability to form skeletal metastases.

## INTRODUCTION

TNF-related apoptosis inducing ligand receptor (TRAIL-R) 1 and 2, both members of the TNF receptor superfamily, are able to induce both apoptotic and non-apoptotic signaling when cross-linked at the plasma membrane by their cognate ligand TRAIL [1–4]. Since TRAIL induces cell death preferentially in tumor cells while sparing most non-transformed cells [5, 6], TRAIL and agonistic TRAIL-R1/-R2-specific antibodies are currently under clinical investigation as potential treatment strategies for different malignancies [7, 8]. Counterintuitively, a variety of tumors overexpress these death receptors, and for some, high expression of primarily TRAIL-R2, but also TRAIL-R1, has been associated with a poor prognosis [9–20]. This observation may reflect the pro-tumoral signaling of membrane-bound TRAIL receptors in response to TRAIL present in the tumor microenvironment. Such signaling after treatment with recombinant TRAIL has been shown to give rise to enhanced motility, invasion and metastatic potential of different tumor cells [4, 21–23]. However, more critical evaluations of immunohistochemical staining revealed mainly intracellular distribution of TRAIL death receptors in tumor tissues (for review see [20]). Such intracellular locations, either in the cytoplasm or in the nucleus, have been mainly regarded as hide-outs for these receptors, representing a strategy for cancer cells to resist TRAIL-mediated apoptosis [24, 25]. Moreover, the intracellular sequestering of TRAIL receptors could potentially represent an escape mechanism of tumor cells from immune surveillance.

A newly described, nuclear-specific function of TRAIL-R2 links high intracellular receptor expression to enhanced malignancy of tumor cells [9]. In this cellular compartment, TRAIL-R2, acting as a negative regulator of let-7 miRNA maturation, increases the expression of the respective targets high mobility group AT-hook 2 (HMGA2) and lin-28 homolog B (Lin28B), and enhances tumor cell proliferation [9]. Thus, both plasma membrane and nuclear TRAIL receptors, although via entirely different mechanisms, may contribute to tumor progression. This raises the question whether the down regulation of TRAIL-R2 expression, instead of its targeting by agonistic molecules, may offer a better therapeutic option for some tumor entities.

Breast cancer incidence is on the rise and is still the most common malignancy among women. Although survival rates have improved over the last decades, one third of patients relapse with distant metastases. Approximately 70% of breast cancer patients in advanced tumor stages will develop metastases in the skeleton, which may cause clinically relevant morbidities such as chronic bone pain, pathological fractures, neurological compression syndromes or severe hypercalcemia [26, 27]. Once distant metastases occur, treatment is mainly palliative in nature. Therefore, significant ongoing research effort is focused on the prevention and accurate treatment of these metastases. Different sets of genes or proteins in tumor cells have been described to predict metastasis to the bone or other organs, though none exclusively satisfied expectations [28, 29]. Moreover, it has recently been postulated that mesenchymal stromal cells in the microenvironment of primary breast tumors preselect carcinoma subclones with a high bone tropism via secretion of stromal cell-derived factor 1 (SDF-1) and insulin-like growth factor 1 (IGF-1) [30]. Interestingly, these clones are characterized by high Src-activity, a known predictor for bone relapse, which potentiates pro-tumoral effects via activation of the PI3K-/AKT pathways upon SDF-1 stimulation [31]. Once colonized to the bone, metastatic cancer cells have the ability to positively influence the bone-degrading activity of osteoclasts via secretion of osteoclastogenic factors or through interactions with mesenchymal stromal cells, impairing physiological bone turnover. As a consequence, large amounts of growth factors are released through the breakdown of bone matrices which in turn promote the growth of tumor cells resulting in a “vicious cycle”.

Immunohistochemical analyses of tissue samples from a large cohort of breast cancer patients detected overexpression of both TRAIL death receptors in tumor tissue and revealed their clear prognostic clinical value [13]. High expression of TRAIL-R1 correlated with the presence of favorable prognostic markers (e.g. better cell differentiation, negative lymph node status). In contrast, high expression of TRAIL-R2 correlated with worse prognostic markers, such as a high Ki67- index, HER2-neu status or metastatic lymph nodes, and decreased survival [13, 14].

In the present study we therefore aimed to evaluate the impact of constitutive high expression of TRAIL-R2 on breast cancer cell biology *in vitro* and *in vivo*, with particular emphasis on its role in the process of bone metastasis. For this purpose, we utilized a highly osteotropic, “bone seeking” variant of the breast cancer cell line MDA-MB-231 (MDA-MB-231-BO), selected from repeated *in vivo* passaging [32]. We demonstrate that the knockdown of TRAIL-R2 in these cells down regulates the levels of bone metastasis-related proteins HMGA2, p-Src and C-X-C chemokine receptor 4 (CXCR4), leads to the re-expression of E-cadherin, and dramatically impairs the capability of cells to metastasize to the bone.

## RESULTS

### Knockdown of TRAIL-R2 inhibits proliferation and migration of an osteotropic MDA-MB-231 variant *in vitro*

**Figure 1:**
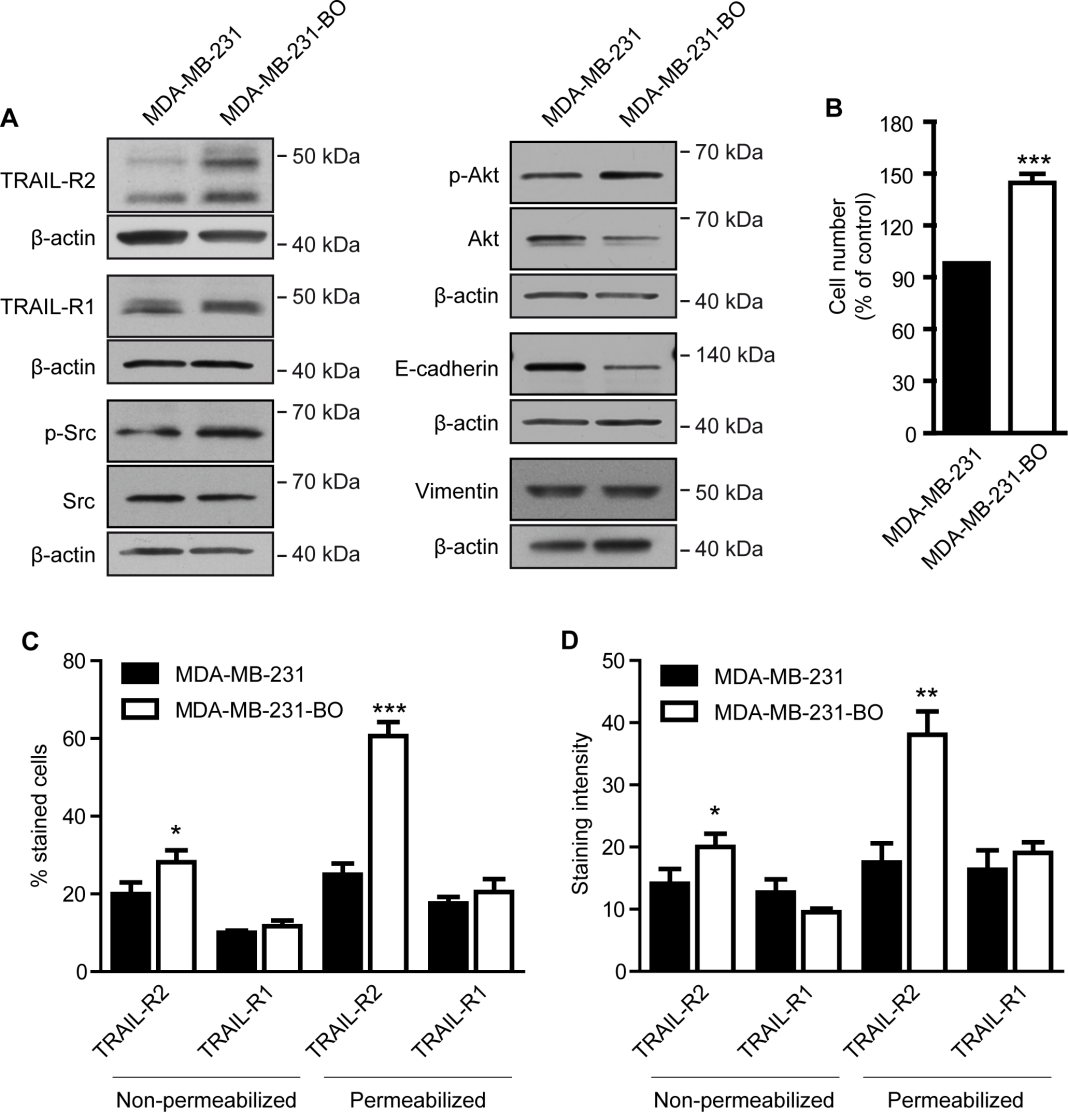
Bone metastatic phenotype of MDA-MB-231 cells is associated with higher expression of TRAIL-R2 and enhanced proliferation Whole cell lysates of MDA-MB-231 and their bone-seeking variant MDA-MB-231-BO were analyzed by Western blot for the expression of depicted proteins **(A)**. As a control for equal gel loading, ß-actin levels were analyzed. **(B)** Proliferation of parental MDA-MB-231 and bone seeking variant were determined 72 h post seeding. Cell surface (non-permeabilized) and total (permeabilized) levels of TRAIL-R1 and R2 were analyzed by FACS and quantified for the percent of cells **(C)** and the staining intensity per cell **(D)** for each death receptor. Graphs represent average values ± SD (*n* = 5) (*p < 0.05, **p < 0.01, ***p < 0.001).

While expression of TRAIL-R2 was shown to correlate with unfavorable prognostic markers in primary breast tumors [13], the impact of TRAIL-R2 in the metastatic behavior of breast cancer cells has not yet been addressed. We therefore first compared the steady state levels of TRAIL-R2 in the parental breast cancer cell line MDA-MB-231 and its bone-seeking variant MDA-MB-231-BO. These osteotropic cells were selected in preclinical mouse model after repeated *in vivo* passages and show the skeleton as the preferential distant site of metastases formation after intracardial injection [32]. As expected for this more aggressive cell line, MDA-MB-231-BO cells exhibit higher levels of the activated kinases Src and Akt, decreased levels of the epithelial to mesenchymal marker (EMT) E-cadherin and increased proliferation, compared to parental cells (Figure 1A and 1B). The levels of the mesenchymal marker vimentin, however, were unchanged. Importantly, the expression of TRAIL-R2 was strongly increased in these cells. FACS analysis (Figure 1C and 1D) confirmed the weak but significant upregulation of TRAIL-R2 at the cell surface which coincided with an increased sensitivity to TRAIL-induced apoptosis (Supplementary Figure 1A). Importantly, these analyses also revealed a strong and highly significant enhancement of total TRAIL-R2 levels. Because FACS analyses showed no significant changes in the expression of TRAIL-R1, either at the cell surface or intracellularly, these results suggest that TRAIL-R2 may play a role in promoting breast cancer bone metastasis.

**Figure 2:**
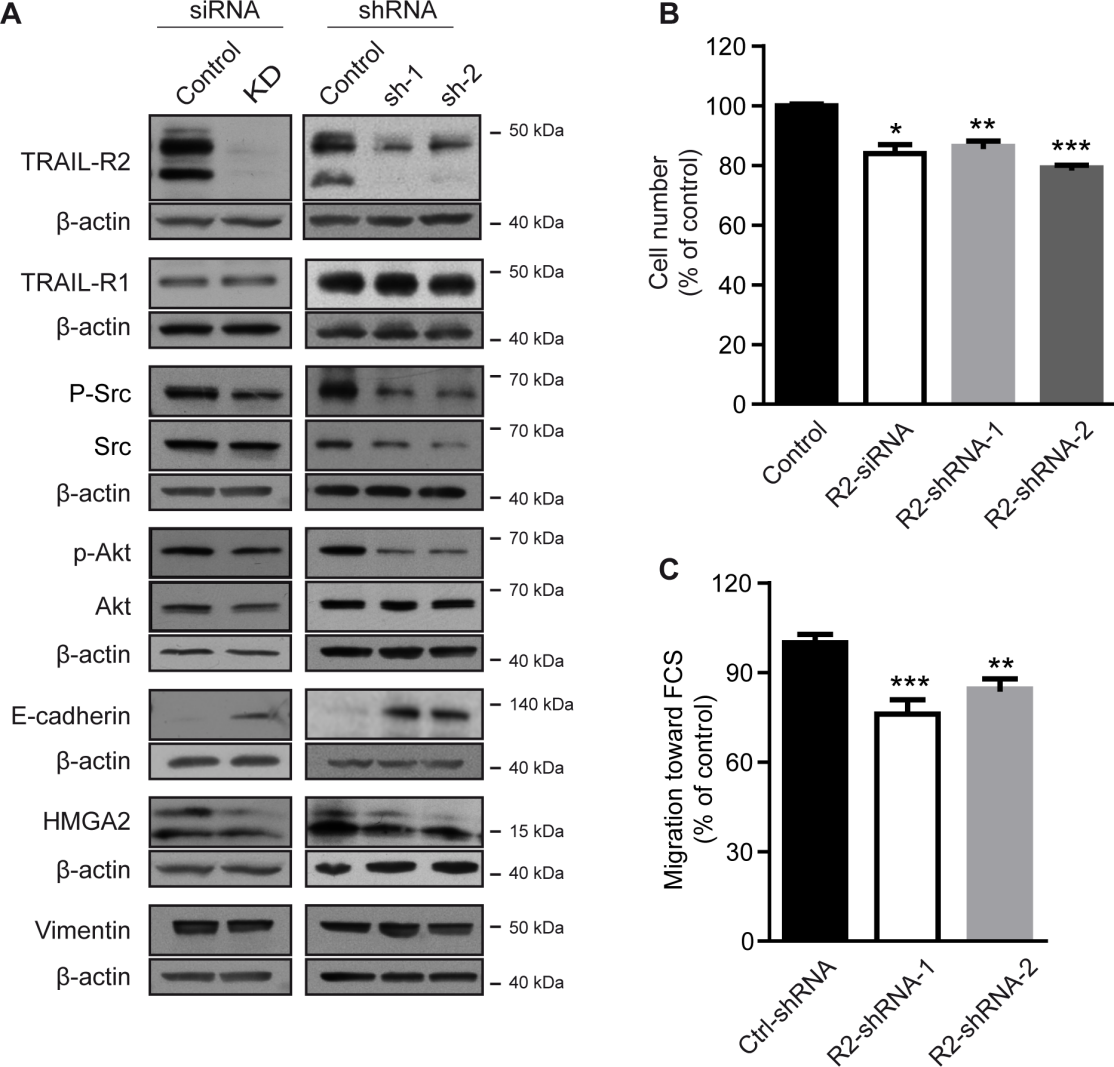
Knockdown of TRAIL-R2 reverses the bone-metastatic signature of MDA-MB-231-BO cells and impairs proliferation and migration TRAIL-R2 was either transiently (siRNA) or stably (shRNA) down regulated in MDA-MB-231-BO cells. Protein levels were determined by Western blotting in whole cell lysates **(A)**. β-actin levels were used as a loading control. **(B)** Proliferation of cells with either transient (siRNA) or stable (shRNA) down regulation of TRAIL-R2 was determined by cell counting 72 h post transfection or 72 h after seeding, respectively, and graphed relative to their individual controls (*n* = 3). **(C)** TRAIL-R2 knockdown cells (R2-shRNA) or control cells (Ctrl-shRNA) were analyzed in regard to their migration capacity towards FCS in trans-well assays. Graphs represent average values ± SD (*n* = 4) (*p < 0.05, **p < 0.01, ***p < 0.001).

To test this hypothesis, expression of TRAIL-R2 in MDA-MB-231-BO cells was knocked down and the resulting phenotype characterized. Modulation of TRAIL-R2, either transiently using siRNA or stably using two different shRNAs, showed substantial decreases in TRAIL-R2 protein levels (Figure 2A), and correspondingly, a decreased sensitivity to TRAIL-induced death (Supplementary Figure 1B). Downregulation of TRAIL-R2 was also associated with reduced levels of p-Akt and p-Src, and increased levels of E-cadherin. Confirming our previous results [9], knockdown of TRAIL-R2 also led to down regulation of HMGA2 and impaired cell proliferation (Figure 2B).

To determine potential changes in metastatic potential, TRAIL-R2 knockdown cells were assessed for impaired migration capacity (Figure 2C). Indeed, cells stably-expressing TRAIL-R2-shRNA showed significant reductions in their ability to migrate towards FCS as determined by the *in vitro* trans-well migration assay in the modified Boyden chamber.

**Figure 3:**
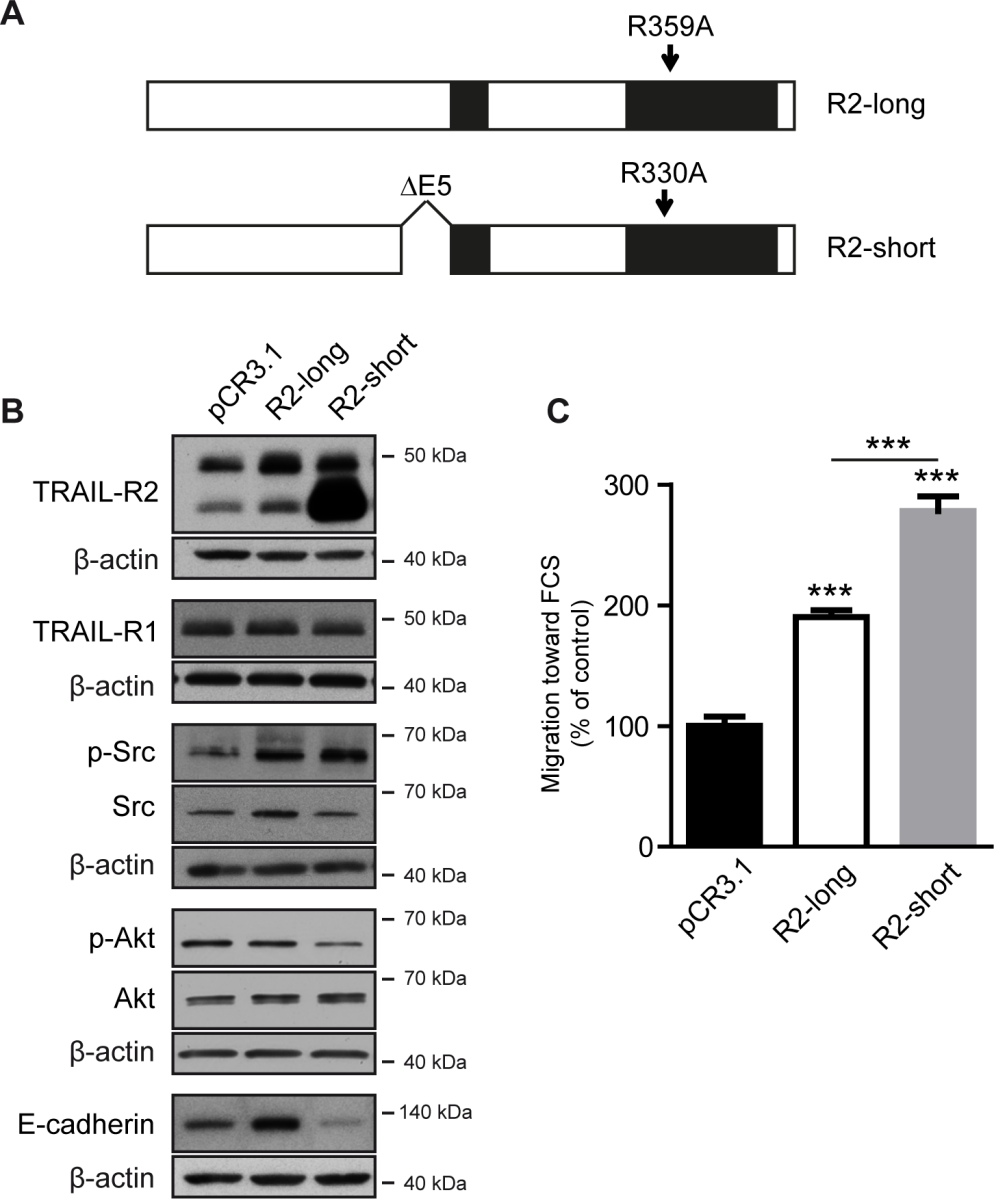
Overexpression of TRAIL-R2 modulates the bone metastatic signature and enhances cell migration MDA-MB-231 cells were stably transfected with the expression vectors coding for the long (R2-long) or short (R2-short) isoforms of TRAIL-R2, both carrying a point mutation in the death domain, or with an empty vector (pCR3.1) **(A)**. Protein levels were determined by Western blotting in whole cell lysates **(B)**. β-actin levels were used as a loading control. Cells were also assessed for cell migration towards FCS **(C)**. Graphs represent average values ± SD (*n* = 4) (**p < 0.01, ***p < 0.001).

Since TRAIL-R2 exists in two isoforms, we further asked which isoform is involved in mediating these effects. We therefore constructed expression vectors for both TRAIL-R2 isoforms (Figure 3A). To avoid the induction of apoptosis due to clustering of the overexpressed death receptors, we introduced a mutation into the death domain (DD) of TRAIL-R2 preventing its interaction with FADD [33]. As shown in Figure 3B, TRAIL-R2 isoforms differentially impact the expression of E-cadherin and the phosphorylation status of Akt. Whereas overexpression of the short TRAIL-R2-isoform led to a clear down regulation of E-cadherin and to diminished Akt activity, the overexpression of the long TRAIL-R2 isoform resulted in clear upregulation of E-cadherin and no changes in Akt activity. In contrast, both isoforms had a similar, potentiating impact on the activity of Src and migration capacity towards FCS (Figure 3C).

### Inhibition of TRAIL-R2 impairs the bone metastatic capacity of MDA-MB-231-BO cells

**Figure 4:**
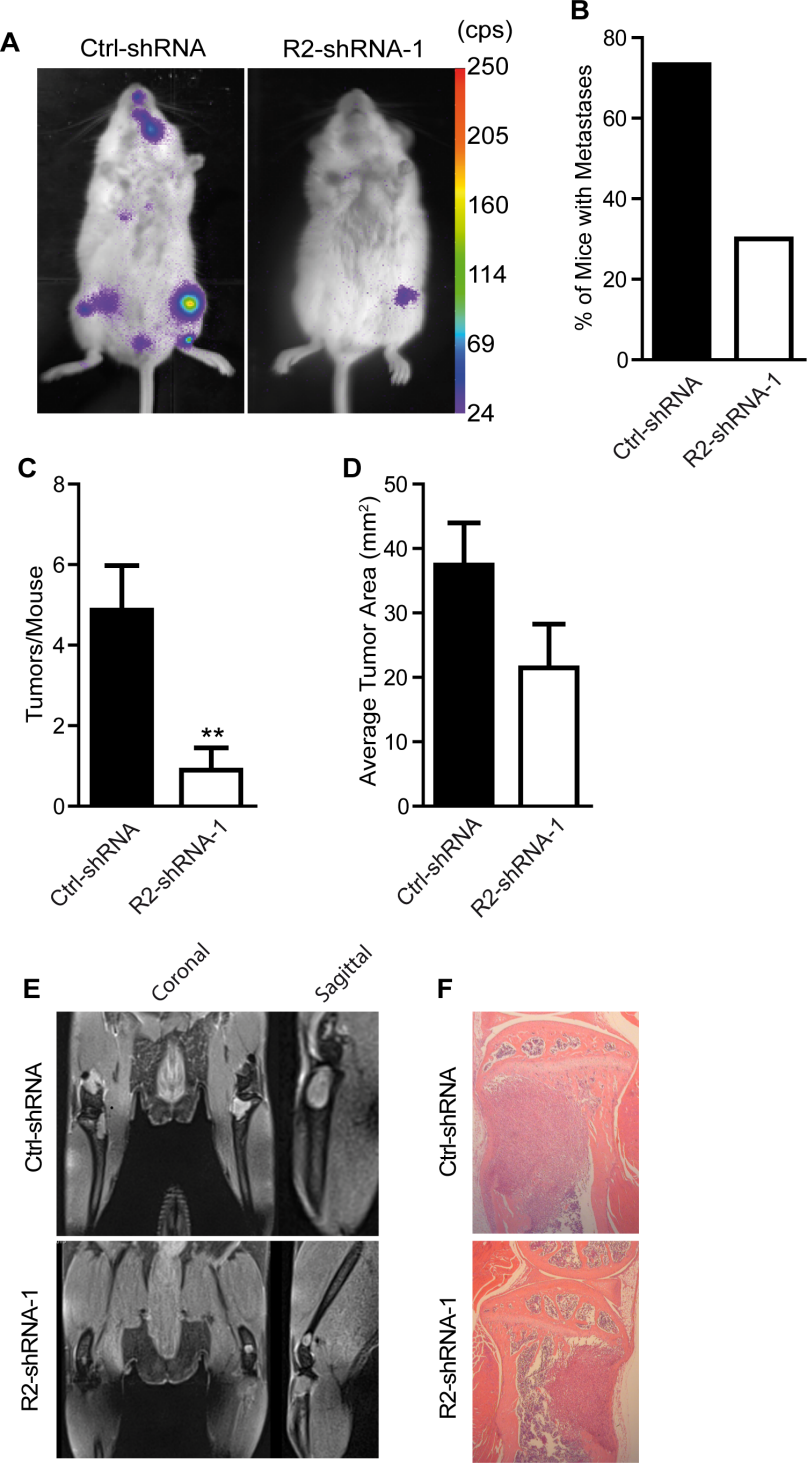
Knockdown of TRAIL-R2 impairs the formation of bone metastasis *in vivo* Control (Ctrl-shRNA) and TRAIL-R2 knockdown cells (R2-shRNA-1) were intracardially-injected and monitored weekly by bioluminescence imaging **(A)**. From bioluminescent imaging, percent of mice forming metastases **(B)**, average number of tumors per mice **(C)** and average tumor area **(D)** were assessed. *In vivo* MRI and *ex vivo* HE-stained histological sections (E and F) were used to confirm that bioluminescent signals originated within the bone and not in the surrounding soft tissue. Graphs represent average values ± SD (*n* = 12) (**p < 0.01).

To analyze the impact of TRAIL-R2 in the process of bone metastases formation and progression, control cells and cells with reduced expression of TRAIL-R2 were injected intracardially into SCID-beige mice and subsequent tumor development was monitored by bioluminescent imaging (Figure 4A). Knockdown of TRAIL-R2 substantially decreased the formation of metastases. While 73% of mice injected with control-shRNA cells developed bone metastases, only 30% of mice injected with TRAIL-R2-shRNA-1 showed detectable bone lesions after reaching the ethical endpoint of 5 weeks (Figure 4B). In addition, mice injected with TRAIL-R2-shRNA-1 cells showed a significant reduction in the average number of tumors per mouse (Figure 4C), as well as a reduction in the average tumor area assessed by bioluminescent signal (Figure 4D), relative to mice injected with control-shRNA cells. Bioluminescent signal observed in the knee region was confirmed to originate from lesions within the bone, and not in the surrounding soft tissue, *in vivo* using magnetic resonance imaging (MRI) and *ex vivo* by histological analyses (Figure 4E and 4F)

**Figure 5:**
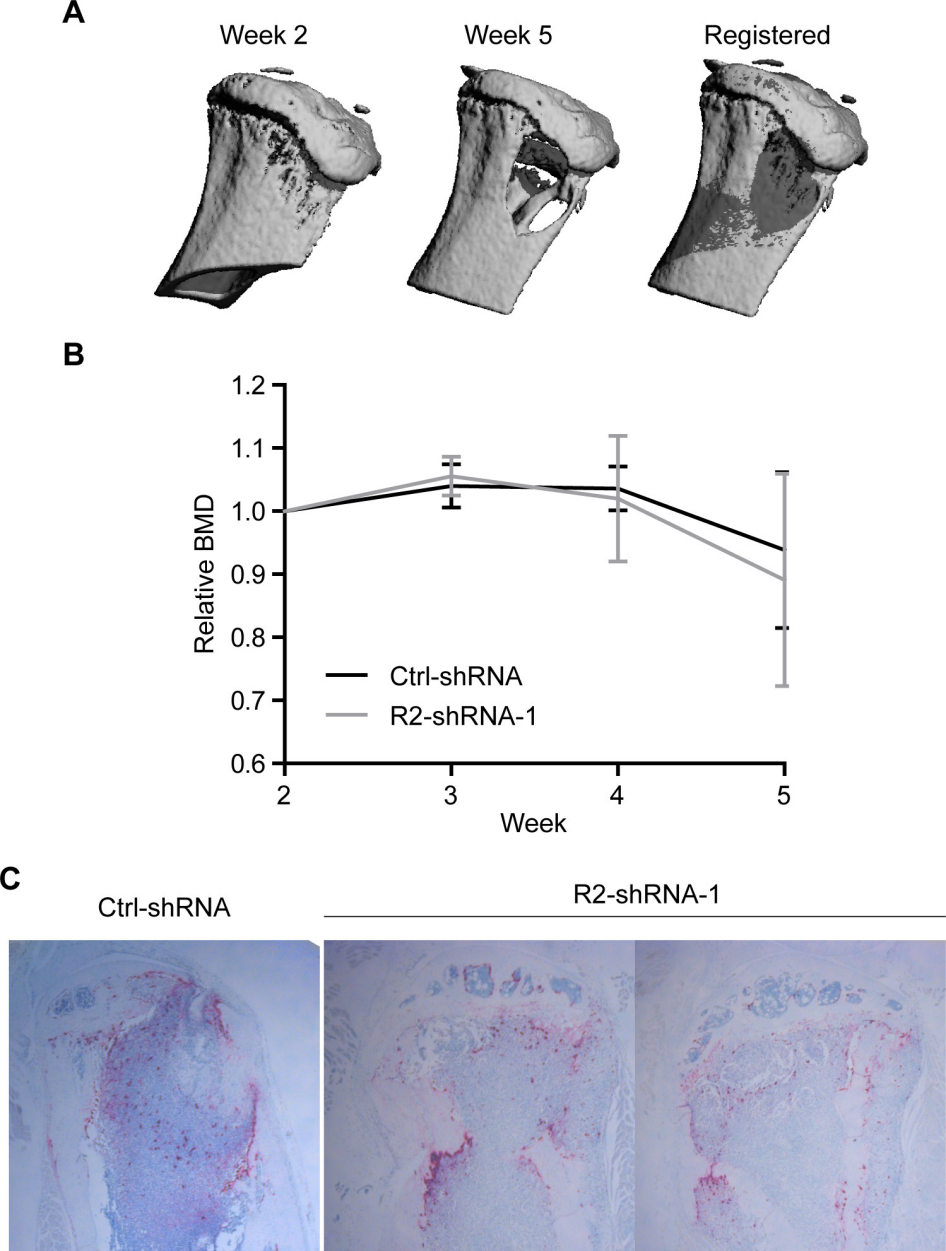
TRAIL-R2 status does not affect osteolysis in MDA-MB-231-BO-derived bone lesions Tumor-burdened mice were imaged weekly by *in vivo* micro-CT. Baseline and follow-up scans were registered **(A)** and relative changes in BMD values were quantified **(B)**. Histological sections were stained for TRAP activity and imaged **(C)**. Images show a representative image of Ctrl-shRNA injected mice and TRAP stained images for both hind limbs which developed tumors in the R2-shRNA group. Graphs represent average values from tumor-burdened limbs ± SD.

To evaluate the magnitude of bone destruction in response to invasive tumor cell growth, mice were subjected to weekly micro-computed tomography (micro-CT) scans to monitor changes in bone mineral density (BMD) (Figure 5A). Tumor-burdened tibiae showed severe osteolysis compared to baseline scans, irrespective of the TRAIL-R2 expression status (Figure 5B). To confirm these data *post mortem*, tartrate-resistant acid phosphatase (TRAP) staining on tumor-bearing bone sections was performed to visualize osteoclast activity. Consistent with micro-CT results, no notable differences were detected in terms of osteoclast activation (Figure 5C), indicating that the intraosseus growth of breast cancer metastases was not dependent on TRAIL-R2 expression levels.

### TRAIL-R2 regulates cellular responsiveness to bone-secreted chemoattractants

**Figure 6:**
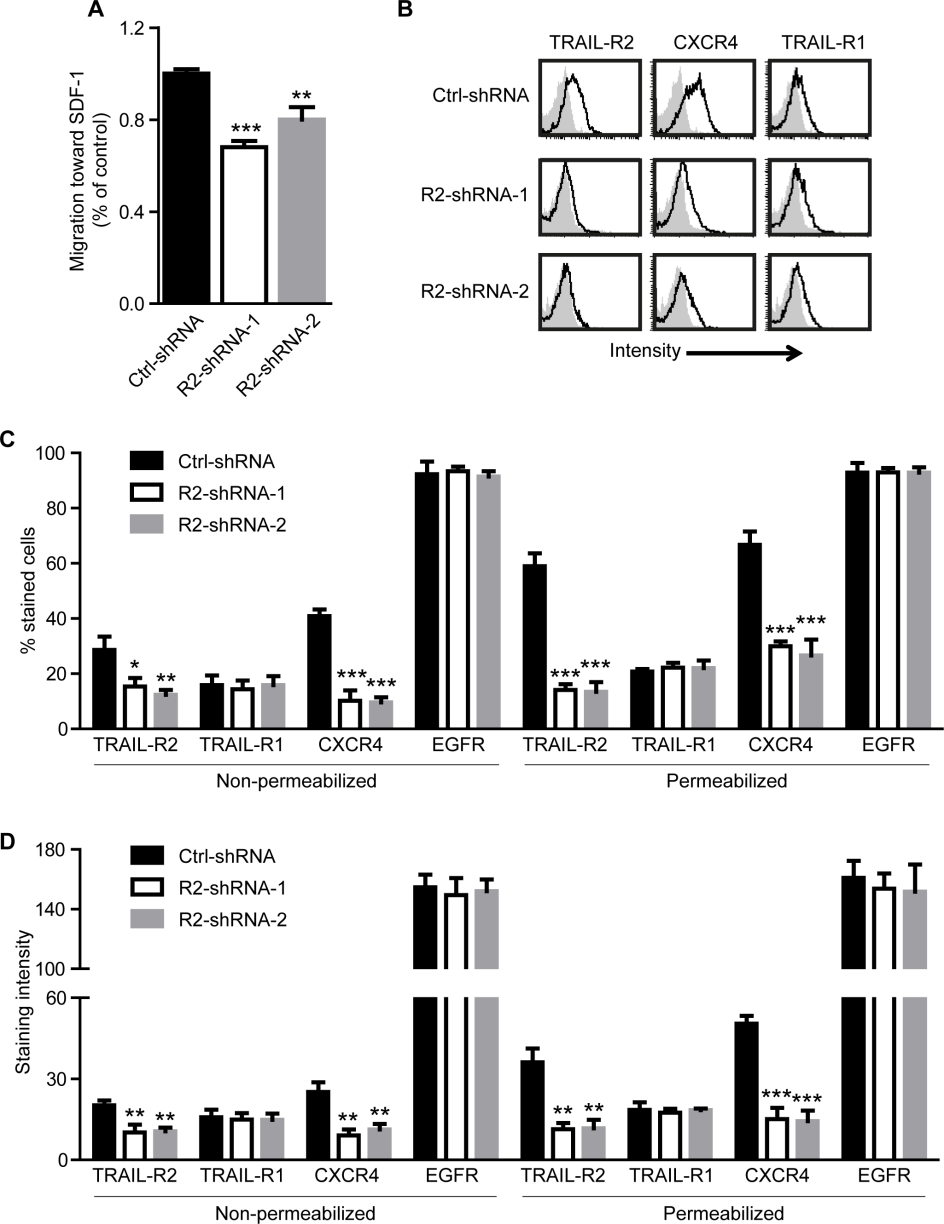
Knockdown of TRAIL-R2 in MDA-MB-231-BO cells downregulates the expression of CXCR4 and inhibits migration towards SDF-1 Control (Ctrl-shRNA) and TRAIL-R2 knockdown cells (R2-shRNA-1 or R2-shRNA-2) were analyzed in regard to their migration capacity towards SDF-1 in a trans-well assay **(A)**. Expression of TRAIL-R1, TRAIL-R2, CXCR4 and EGFR was assessed by flow cytometry on non-permeabilized and permeabilized cells **(B)** and percent of stained cells **(C)**, as well as staining intensities per cell **(D)**, relative to non-specific antibody controls, were quantified. Graphs represent average values ± SD. (A: *n* = 4, B-D: *n* = 3) (*p < 0.05, **p < 0.01 ***p < 0.001).

We found that inhibition of TRAIL-R2 expression in osteotropic breast cancer cells dramatically reduced their capability to form skeletal metastases after intracardiac injection. However, once established, the metastatic growth, as well as the osteolytic activity, was independent of the TRAIL-R2 expression status. This strongly suggests a role for TRAIL-R2 in the regulation of the bone homing capacity of these cells. To test this hypothesis, cells were analyzed for their ability to migrate towards SDF-1, a major bone marrow-secreted chemoattractant for metastatic cancer cells [34]. Using an *in vitro* migration assay in the modified Boyden Chamber, we found that knockdown of TRAIL-R2 indeed significantly decreased the migration capacity of the cells towards SDF-1 (Figure 6A).

Searching for the mechanism explaining this phenomenon, we analyzed the plasma membrane and overall expression levels of the receptor for SDF-1, CXCR4, in control and TRAIL-R2-knockdown cells. For this purpose, non-permeabilized and permeabilized cells were stained for TRAIL-R2 and CXCR4, along with epidermal growth factor receptor (EGFR), frequently expressed at high levels in metastatic breast cancer cells, and TRAIL-R1 (Figure 6B). Along with significant reductions in TRAIL-R2, TRAIL-R2-knockdown cells also showed significant reductions in the percent of cells stained and intensity of staining per cell observed for CXCR4 (Figure 6C and 6D, Supplementary Table 1). This decrease was specific for CXCR4, as no significant changes were observed for TRAIL-R1 or EGFR.

**Figure 7:**
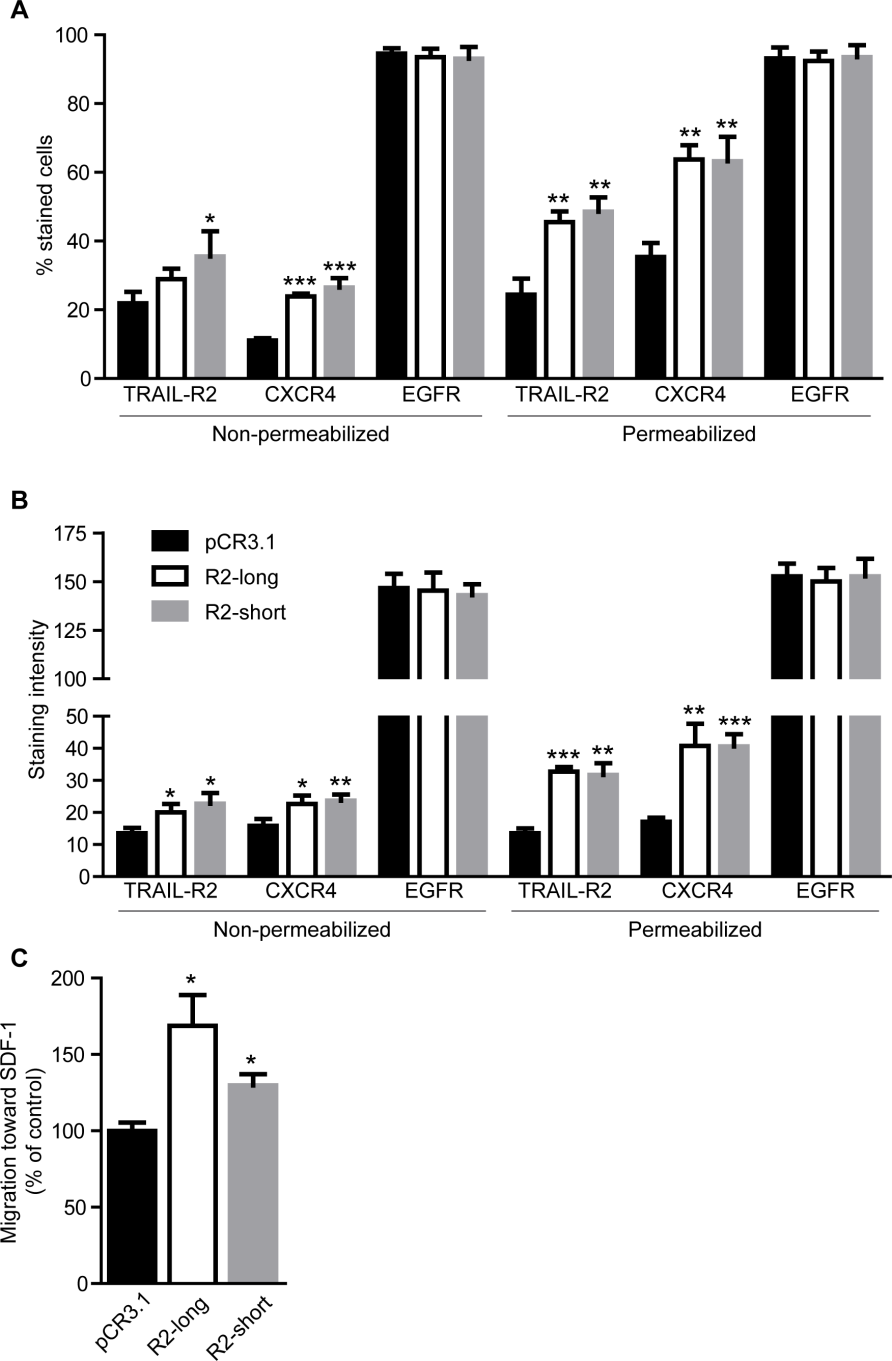
Overexpression of TRAIL-R2 in MDA-MB-231 cells upregulates the expression of CXCR4 and enhances migration towards SDF-1 Expression of TRAIL-R2, CXCR4 and EGFR in control cells (pCR3.1) and cells overexpressing the long (R2-long) or short (R2-short) isoforms was assessed by flow cytometry in non-permeabilized and permeabilized cells and percent of stained cells **(A)** and staining intensities per cell **(B)**, relative to non-specific antibody controls, were quantified. **(C)** Cells were analyzed in regard to their migration capacity towards SDF-1 in a trans-well assay. Graphs represent average values ± SD. (A–B: *n* = 3, C: *n* = 4) (*p < 0.05, **p < 0.01 ***p < 0.001).

Importantly, while EGFR remained unchanged, overexpression of either TRAIL-R2-short or TRAIL-R2-long isoform in parental MDA-MB-231 cells strongly and specifically enhanced the plasma membrane and overall expression levels of CXCR4 as shown by FACS analyses (Figure 7A and 7B, Supplementary Table 2). In accordance, overexpression of either isoform resulted in strongly enhanced migration towards SDF-1 (Figure 7C).

## DISCUSSION

Prompted by the observations that high intracellular expression of TRAIL-R2 correlates with poor clinical prognostic parameters [13, 14], we set out to explore the role of this particular member of the death receptor family in the malignancy of breast cancer cells, particularly in their capacity to metastasize to the bone. The clear upregulation of TRAIL-R2 levels in bone-seeking MDA-MB-231-BO cells, as compared with parental cells, suggests a potential relevance of TRAIL-R2 in the process of bone metastasis formation and/or progression.

In agreement with our previous data for the parental cell line MDA-MB-231, as well as for other tumor cell lines [9], knockdown of TRAIL-R2 resulted in decreased expression of HMGA2, and correspondingly, decreased proliferation. These effects were accompanied by the upregulation of the EMT marker E-cadherin and impaired cell migration. *In vivo*, knockdown of TRAIL-R2 drastically inhibited initial bone metastasis formation, reducing both the frequency and number of formed metastases. However, once tumors were established, no noticeable differences in tumor-associated bone loss or osteoclast activity were observed. Thus, we hypothesize that knockdown of TRAIL-R2 inhibits bone homing and early colonization to the bone marrow, rather than tumor growth within the bone marrow niche. Once these cells begin to grow in the bone marrow and enter the “vicious cycle” of bone degradation by osteoclasts, leading to stimulation of tumor cell activity via the release of growth factors from the bone matrix, inhibition of TRAIL-R2 expression shows a less profound effect on tumor progression.

EMT, with downregulation of E-cadherin expression as a hallmark [35], is regarded as a prerequisite for the initiating steps of the metastatic cascade involving detachment of the cells from the primary tumor, intravasation, survival in the circulation, extravasation and early colonization by the establishment of the dormant micrometastases in the microenvironment of the distant organ. For successful development of macrometastases in this secondary tumor site, however, the necessity for the switch back to the epithelial phenotype (mesenchymal to epithelial transition) (MET) was postulated for bone colonization by metastatic breast cancer cells [28]. Consistent with this model, and with well documented changes in skeletal metastasis of MDA-MB-231 cells by modulation of E-cadherin [36], knockdown of TRAIL-R2 cells in the osteotropic variant of these cells, which resulted in an upregulation of E-cadherin, dramatically impaired their bone metastatic capacity.

Along with a general impairment in cell migration, the knockdown of TRAIL-R2 expression also resulted in reduced migration towards the bone marrow-derived cytokine SDF-1. SDF-1 is regarded as the key cytokine driving bone chemoattractance of breast cancer cells. Interestingly, SDF-1-responsive osteotropic tumor cells seem to be pre-selected in the primary tumor by SDF-1 produced by mesenchymal cells present in the primary tumor stroma [30]. The observed inhibition of SDF-1-induced migration of TRAIL-R2 knockdown cells was most likely due to the significantly decreased levels of CXCR4 in these cells, previously shown to play a critical role in breast cancer metastasis [29, 37]. Importantly, recent meta-analysis validated CXCR4 as a prognostic marker for breast cancer [38] and demonstrated the association of high CXCR4 expression with lymph node status, distant metastasis, and poor overall and disease free survival. Although the mechanism of TRAIL-R2-mediated regulation of CXCR4 expression is currently unknown, it is likely that its nuclear function as a regulator of let-7 maturation [9] may play a role. This hypothesis is supported by recent data which demonstrate that let-7, due to its ability to decrease the levels of HMGA2 resulting in the down regulation of CXCR4, inhibits the formation of breast cancer bone metastases [39]. However, since tumor cells may produce and secrete their own TRAIL, we cannot rule out the role of plasma membrane-bound TRAIL-R2, which is known to induce signaling pathways promoting cell invasion and metastasis following TRAIL treatment [42]. Further *in vitro* and *in vivo* experiments performed in the presence of agents neutralizing endogenous (tumor cell-derived or mouse) TRAIL will help to understand the cellular function of TRAIL-R2 in its different cellular compartments as a promoter of skeletal metastasis of breast cancer cells.

Besides HMGA2 and CXCR4, the expression of several other genes have also been shown to be important in the bone metastatic phenotype of cancer cells [39, 40]. Analysis of protein levels between control cells and TRAIL-R2-knockdown cells detected decreased levels of p-Src and p-Akt. Both factors have been shown to play a major role in early colonization of breast cancer cells to the bone matrix [28, 31]. Zhang et al hypothesized that Src-activity potentiates pro-tumoral SDF-1 signaling via enhancement of the PI3K/Akt-pathway [31].

However, several important questions remain open. First, the majority of breast cancer patients bearing skeletal metastases suffer from estrogen and progesterone receptor-positive cancer. Therefore, further studies are needed to evaluate the impact of TRAIL-R2 in these more common types of breast cancer cells. Second, it cannot be ruled out that, in addition to the novel pro-tumorigenic, nuclear TRAIL-R2 function, the previously described pro-tumorigenic plasma membrane TRAIL-R2 signaling may also contribute to the observed effects. Immunohistological studies have revealed a correlation of high intracellular expression of TRAIL-R2, and coinciding almost undetectable plasma membrane expression, with worse patient prognosis. Careful re-evaluation of the histopathological data in respect to intracellular localization of this receptor and its correlation with clinical parameters will help to evaluate the role of each particular TRAIL-R2 population in bone metastasis of breast cancer cells. Recent discoveries of the novel TRAIL-R2 function as a regulator of miRNA-maturation in the nucleus [9], and as ER-stress-mediated, ligand-independent inducer of apoptosis in the cytoplasm [41], strongly demands further studies on the role of TRAIL death receptors in non-canonical localizations, and in particular, on the mechanisms of its intracellular trafficking.

Importantly, our overexpression experiments revealed potentially differential and also overlapping functions of short and long TRAIL-R2 isoforms. Whereas overexpression of the short TRAIL-R2 isoform led to the downregulation of E-cadherin, overexpression of the long isoform clearly upregulated its cellular levels. However, both isoforms consistently upregulated the expression of CXCR4, potentiated the activity of Src and strongly enhanced cell migration. Further work is required to fully understand how these different isoforms differentially affect levels of these proteins.

In summary, we have uncovered a surprising mechanistic relevance of TRAIL-R2 in the metastatic behavior of breast cancer cells. We show that knockdown of TRAIL-R2 leads to a down regulation of HMGA2, p-Src, p-Akt and CXCR4 and increased expression of the epithelial marker E-cadherin. These cellular changes translate to a strongly diminished occurrence of skeletal metastases *in vivo*, and in turn may explain the correlation of TRAIL-R2 expression with unfavorable prognostic parameters in clinical breast cancer samples [13]. Our results suggest that targeted therapies aimed at decreasing the levels of TRAIL-R2 in primary breast carcinomas and/or circulating tumor cells may represent a promising therapeutic strategy for reducing the risk of breast cancer patients to relapse with bone metastases. In addition, future studies may reveal TRAIL-R2 status to have a prognostic or predictive marker for high risk patients of developing skeletal events.

## MATERIALS AND METHODS

### Cell lines, culture conditions and generation of stable clones

The bone-seeking variant of MDA-MB-231 breast cancer cells MDA-MB-231-BO was kindly provided by Gabri van der Pluijm (Leiden University Medical Center, Netherlands). These cells were isolated from murine tibia metastases after repeated *in vivo* passages following intracardial injections of cell populations resulting in a cell clone with the capability to metastasize to the bone at high frequencies [32]. Cells were cultured in RPMI 1640 media supplemented with 10% FCS, 1 mM GlutaMAX and 1 mM sodium pyruvate (LifeTechnologies, Darmstadt, Germany). For transient knockdown of TRAIL-R2, cells were transfected with On-Target*plus^®^* smart pool siRNAs L-004448–00 (Thermo Scientific Dharmacon, Epsom, UK) using Lipofectamine2000 (Life Technologies). As a control, On-Target*plus^®^* non-targeting pool D-001810–10 was used (Thermo Scientific Dharmacon). To obtain clone pools with stable knockdown of TRAIL-R2, cells were transduced with the GIPZ lentiviral shRNAmir vectors for TRAIL-R2 and non-silencing control (Open Biosystems, Germany; CloneID: TRAIL-R2-shRNA-1: V2LHS_16711, TRAIL-R2-shRNA-2: V3LHS_328891) and selected with 1 μg/ml puromycin.

Isoforms of TRAIL-R2, were generated from full length TRAIL-R2 cloned in the pCR3.1 vector (kindly provided by Pascal Schneider, University of Lausanne, Switzerland using primers CCTGGGAGCCGCTCATGGCGAAGTTGGGC CTCATGGAC (introducing a point mutation in the DD), CATCGAATGTGTCCACAAAGAATCA GGCATCATCATAG (R2-short) and the QuickChange II site-directed mutagenesis kit (Agilent Technologies, La Jolla, CA, USA) as outlined by the manufacturer. All constructs were verified by sequencing.

### Protein expression studies

Whole cell lysates (10 μg per lane) were analyzed by Western blotting as previously described [43]. Primary antibodies used were purchased from: ProScience/Axxora, Lörrach, Germany (anti-TRAIL-R2); Cell Signaling, Schwalbach, Germany (HMGA2, p-Src, Src, p-Akt, Akt,); R&D systems, Minneapolis, MN, USA (E-cadherin); Sigma-Aldrich, St. Louis, MO, USA (β-actin).

Protein levels were additionally assessed using FACS analysis. Briefly, 5 × 10^4^ cells were seeded in 96-well plates and non-permeabilized or fixed and permeabilized (1% formalin, 30 min, 4^o^C, washing, 0.1% Tween, 30 min, 4^o^C, 2x washing) cells were incubated for 30 min at 4^o^C with primary antibodies against CXCR4 (BD Bioscience, San Jose, CA, USA), EGFR (AnaSpec Inc, Fremont, CA, USA), TRAIL-R2 or TRAIL-R1 (HS201 and HS101 both kindly provided by Henning Walczak, Imperial College, London). After washing (PBS, 1% BSA), cells were incubated with APC-labeled secondary antibody (Jackson ImmunoResearch, Newmarket, UK) (30 min, 4^o^C). Analysis was performed with a FACS Calibur and the CellQuest program (BD Bioscience).

### Proliferation and migration assays

To compare cell proliferation, 2.0 × 10^5^ cells were seeded in 6-well plates and grown for 72 h. Cells were detached with Accutase^®^ (GE Healthcare, Little Chalfont, UK), then counted using an automated cell counter (Cellometer Auto T4; Nexcelom Bioscience, Lowrence, USA). To study cell migration, 24-well plate inserts with 8.0 μm pore size polycarbonate membranes (Corning Incorporation, NY, USA), were seeded with 2 × 10^5^ cells. Prior to seeding, cells were starved for 24 h in medium with FCS reduced to 0.5%. The lower chamber was filled with medium containing either 10% FCS or 100 ng/ml SDF-1α (Peprotech, Hamburg, Germany). After 21 h, trans-migrated cells were counted.

### Animal experiments

5–6 week old female, SCID Beige mice were purchased from Charles River (Wilmington, MA). All animals were kept in a temperature and humidity-controlled environment with a 12 h light/dark cycle and access to food and water *ad libitum*. Experiments were carried out in accordance with local authorities (V312–7224.121(75–5/12)). Mice were anesthetized with intra-peritoneal injection of 80 mg/kg ketamine (Aveco Pharmaceutical, IA) and 0.5 mg/kg dorbene (Pfizer, Berlin, Germany). 1.4×10^5^ MDA-MB-231-BO stably transfected cells with control shRNA or shRNA-1 against TRAIL-R2 were suspended in 100 μl 1X PBS and injected into the left ventricle of anesthetized animals under sonographic guidance (*n* = 15/group).

### *In vivo* imaging

Anesthetized mice received 150 mg/kg D-luciferin substrate (Sigma-Aldrich, Munich Germany) injected into the intra-peritoneal space after administration of anesthesia. Dorsal and ventral views were imaged weekly for all mice using the NightOwl planar imaging system (Berthold Technologies, Bad Wildbad, Germany). Tumor bioluminescence was quantified using the Indigo software.

### Magnetic resonance imaging

Magnetic resonance imaging (MRI) measurements were performed at a magnetic field strength of 7 T (ClinScan, Bruker Biospin, Ettlingen, Germany) using a 4-channel phased-array coil for signal reception and a birdcage resonator (inner diameter 70 mm) for excitation (Bruker Biospin). T2-weighted images were obtained by a 2D-turbo-spin-echo sequence (TR/TE 3430/8.9 ms, 4 echoes, resolution 130 × 130 μm, slice thickness 300 μm, 30 slices) and oriented parallel and perpendicular to the mouse leg. Proton density weighted images were acquired by a spoiled 3D-gradient-echo sequence (TR/TE 30/1.1 ms, flip angle 5°, matrix size 192 × 108 × 192) with a spatial resolution of 130 × 130 × 140 μm^3^.

### Micro-CT analysis

Changes in tibial BMD resulting from tumor formation were quantified by *in vivo* micro-computed tomography (micro-CT) as previously described [44]. Anesthetized mice were placed in full-body holders and the tibiae aligned by visual inspection. Weekly scans were made using a vivaCT 40 micro-CT (ScancoMedical, Brüttisellen, Switzerland) at an isotropic voxel size of 19 μm (70 kVp, 114 μA, 250 ms integration time, 1000 projections on 180° 2048 CCD detector array, cone-beam reconstruction). A 140-slice (2.45 mm) volume of interest (VOI), beginning at the most proximal slice of the tibial epiphysis and extending into the metaphyseal region was defined in the baseline scan of each animal using an automated method to draw contours around the periosteal surface [45]. Baseline VOIs were transferred to the follow-up scans using an image registration approach to insure analysis of consistent VOIs at each time point [45]. BMD within the VOI was calculated from the greyscale micro-CT images (Image Processing Language v5.15, ScancoMedical, Brüttisellen, Switzerland).

### Histology

Tibiae containing metastases detectable by bioluminescence were collected and fixed in 10% PBS-buffered formalin for 1 week and subsequently decalcified in PBS-buffered 0.5 M EDTA. Limbs were washed in water, embedded in paraffin and 5 μm sections were prepared. Slices were stained with H&E to distinguish tumor cells from normal bone marrow. To visualize osteoclasts, sections were stained for TRAP activity [46].

### Statistical analyses

All statistical analyses were conducted using Prism (version 5, GraphPad Software, CA). Comparison between groups was made using unpaired *t*-tests. P values of < 0.05 were considered to be statistically significant.

## SUPPLEMENTARY FIGURE AND TABLES



## References

[1] Pan G, O'Rourke K, Chinnaiyan AM, Gentz R, Ebner R, Ni J, Dixit VM (1997). The receptor for the cytotoxic ligand TRAIL. Science.

[2] Screaton GR, Mongkolsapaya J, Xu XN, Cowper AE, McMichael AJ, Bell JI (1997). TRICK2, a new alternatively spliced receptor that transduces the cytotoxic signal from TRAIL. Current biology: CB.

[3] Walczak H, Degli-Esposti MA, Johnson RS, Smolak PJ, Waugh JY, Boiani N, Timour MS, Gerhart MJ, Schooley KA, Smith CA, Goodwin RG, Rauch CT (1997). TRAIL-R2: a novel apoptosis-mediating receptor for TRAIL. The EMBO journal.

[4] Azijli K, Weyhenmeyer B, Peters GJ, de Jong S, Kruyt FAE (2013). Non-canonical kinase signaling by the death ligand TRAIL in cancer cells: discord in the death receptor family. Cell Death Differ.

[5] Ashkenazi A, Pai RC, Fong S, Leung S, Lawrence DA, Marsters SA, Blackie C, Chang L, McMurtrey AE, Hebert A, DeForge L, Koumenis IL, Lewis D, Harris L, Bussiere J, Koeppen H (1999). Safety and antitumor activity of recombinant soluble Apo2 ligand. The Journal of clinical investigation.

[6] Walczak H, Miller RE, Ariail K, Gliniak B, Griffith TS, Kubin M, Chin W, Jones J, Woodward A, Le T, Smith C, Smolak P, Goodwin RG, Rauch CT, Schuh JC, Lynch DH (1999). Tumoricidal activity of tumor necrosis factor-related apoptosis-inducing ligand *in vivo*. Nature medicine.

[7] Micheau O, Shirley S, Dufour F (2013). Death receptors as targets in cancer. British journal of pharmacology.

[8] Lemke J, von Karstedt S, Zinngrebe J, Walczak H (2014). Getting TRAIL back on track for cancer therapy. Cell Death Differ.

[9] Haselmann V, Kurz A, Bertsch U, Hubner S, Olempska-Muller M, Fritsch J, Hasler R, Pickl A, Fritsche H, Annewanter F, Engler C, Fleig B, Bernt A, Roder C, Schmidt H, Gelhaus C (2014). Nuclear death receptor TRAIL-R2 inhibits maturation of let-7 and promotes proliferation of pancreatic and other tumor cells. Gastroenterology.

[10] Bavi P, Prabhakaran SE, Abubaker J, Qadri Z, George T, Al-Sanea N, Abduljabbar A, Ashari LH, Alhomoud S, Al-Dayel F, Hussain AR, Uddin S, Al-Kuraya KS (2010). Prognostic significance of TRAIL death receptors in Middle Eastern colorectal carcinomas and their correlation to oncogenic KRAS alterations. Molecular cancer.

[11] Gallmeier E, Bader DC, Kriegl L, Berezowska S, Seeliger H, Goke B, Kirchner T, Bruns C, De Toni EN (2013). Loss of TRAIL-receptors is a recurrent feature in pancreatic cancer and determines the prognosis of patients with no nodal metastasis after surgery. PloS one.

[12] Sträter J, Hinz U, Walczak H, Mechtersheimer G, Koretz K, Herfarth C, Möller P, Lehnert T (2002). Expression of TRAIL and TRAIL receptors in colon carcinoma: TRAIL-R1 is an independent prognostic parameter. Clinical cancer research: an official journal of the American Association for Cancer Research.

[13] Ganten TM, Sykora J, Koschny R, Batke E, Aulmann S, Mansmann U, Stremmel W, Sinn HP, Walczak H (2009). Prognostic significance of tumour necrosis factor-related apoptosis-inducing ligand (TRAIL) receptor expression in patients with breast cancer. J Mol Med.

[14] McCarthy MM, Sznol M, DiVito KA, Camp RL, Rimm DL, Kluger HM (2005). Evaluating the Expression and Prognostic Value of TRAIL-R1 and TRAIL-R2 in Breast Cancer. Clinical cancer research: an official journal of the American Association for Cancer Research.

[15] Kuijlen JM, Mooij JJ, Platteel I, Hoving EW, van der Graaf WT, Span MM, Hollema H, den Dunnen WF (2006). TRAIL-receptor expression is an independent prognostic factor for survival in patients with a primary glioblastoma multiforme. Journal of neuro-oncology.

[16] Elrod HA, Fan S, Muller S, Chen GZ, Pan L, Tighiouart M, Shin DM, Khuri FR, Sun SY (2010). Analysis of death receptor and caspase-8 expression in primary and metastatic head and neck squamous cell carcinoma and their prognostic impact. PloS one.

[17] Maduro JH, Noordhuis MG, ten Hoor KA, Pras E, Arts HJ, Eijsink JJ, Hollema H, Mom CH, de Jong S, de Vries EG, de Bock GH AG (2009). The prognostic value of TRAIL and its death receptors in cervical cancer. International journal of radiation oncology, biology, physics.

[18] Cooper WA, Kohonen-Corish MR, Zhuang L, McCaughan B, Kennedy C, Screaton G, Sutherland RL, Lee CS (2008). Role and prognostic significance of tumor necrosis factor-related apoptosis-inducing ligand death receptor DR5 in nonsmall-cell lung cancer and precursor lesions. Cancer.

[19] Spierings DC, de Vries EG, Timens W, Groen HJ, Boezen HM, de Jong S (2003). Expression of TRAIL and TRAIL death receptors in stage III non-small cell lung cancer tumors. Clinical cancer research: an official journal of the American Association for Cancer Research.

[20] Bertsch U, Roder C, Kalthoff H, Trauzold A (2014). Compartmentalization of TNF-related apoptosis-inducing ligand (TRAIL) death receptor functions: emerging role of nuclear TRAIL-R2. Cell death & disease.

[21] Ishimura N, Isomoto H, Bronk SF, Gores GJ (2006). Trail induces cell migration and invasion in apoptosis-resistant cholangiocarcinoma cells. American journal of physiology Gastrointestinal and liver physiology.

[22] Trauzold A, Siegmund D, Schniewind B, Sipos B, Egberts J, Zorenkov D, Emme D, Roder C, Kalthoff H, Wajant H (2006). TRAIL promotes metastasis of human pancreatic ductal adenocarcinoma. Oncogene.

[23] Hoogwater FJ, Nijkamp MW, Smakman N, Steller EJ, Emmink BL, Westendorp BF, Raats DA, Sprick MR, Schaefer U, Van Houdt WJ, De Bruijn MT, Schackmann RC, Derksen PW, Medema JP, Walczak H, Borel Rinkes IH (2010). Oncogenic K-Ras turns death receptors into metastasis-promoting receptors in human and mouse colorectal cancer cells. Gastroenterology.

[24] Chen JJ, Shen JH-C, Rivera Rosado L, Zhang Y, Di X, Zhang B (2012). Mislocalization of death receptors correlates with cellular resistance to their cognate ligands in human breast cancer cells. Oncotarget.

[25] Kojima Y, Nakayama M, Nishina T, Nakano H, Koyanagi M, Takeda K, Okumura K, Yagita H (2011). Importin beta1 protein-mediated nuclear localization of death receptor 5 (DR5) limits DR5/tumor necrosis factor (TNF)-related apoptosis-inducing ligand (TRAIL)-induced cell death of human tumor cells. The Journal of biological chemistry.

[26] Coleman RE (2001). Metastatic bone disease: clinical features, pathophysiology and treatment strategies. Cancer treatment reviews.

[27] Roodman GD (2012). Genes associate with abnormal bone cell activity in bone metastasis. Cancer metastasis reviews.

[28] Nutter F, Holen I, Brown HK, Cross SS, Evans CA, Walker M, Coleman RE, Westbrook JA, Selby PJ, Brown JE, Ottewell PD (2014). Different molecular profiles are associated with breast cancer cell homing compared with colonisation of bone: evidence using a novel bone-seeking cell line. Endocrine-related cancer.

[29] Kang Y, Siegel PM, Shu W, Drobnjak M, Kakonen SM, Cordon-Cardo C, Guise TA, Massague J (2003). A multigenic program mediating breast cancer metastasis to bone. Cancer cell.

[30] Zhang XH, Jin X, Malladi S, Zou Y, Wen YH, Brogi E, Smid M, Foekens JA, Massague J (2013). Selection of bone metastasis seeds by mesenchymal signals in the primary tumor stroma. Cell.

[31] Zhang XH, Wang Q, Gerald W, Hudis CA, Norton L, Smid M, Foekens JA, Massague J (2009). Latent bone metastasis in breast cancer tied to Src-dependent survival signals. Cancer cell.

[32] Wetterwald A, van der Pluijm G, Que I, Sijmons B, Buijs J, Karperien M, Lowik CW, Gautschi E, Thalmann GN, Cecchini MG (2002). Optical imaging of cancer metastasis to bone marrow: a mouse model of minimal residual disease. The American journal of pathology.

[33] McDonald ER, Chui PC, Martelli PF, Dicker DT, El-Deiry WS (2001). Death domain mutagenesis of KILLER/DR5 reveals residues critical for apoptotic signaling. The Journal of biological chemistry.

[34] Palacios-Arreola MI, Nava-Castro KE, Castro JI, Garcia-Zepeda E, Carrero JC, Morales-Montor J (2014). The Role of Chemokines in Breast Cancer Pathology and Its Possible Use as Therapeutic Targets. Journal of immunology research.

[35] Lee JM, Dedhar S, Kalluri R, Thompson EW (2006). The epithelial-mesenchymal transition: new insights in signaling, development, and disease. The Journal of cell biology.

[36] Mbalaviele G, Dunstan CR, Sasaki A, Williams PJ, Mundy GR, Yoneda T (1996). E-cadherin expression in human breast cancer cells suppresses the development of osteolytic bone metastases in an experimental metastasis model. Cancer research.

[37] Muller A, Homey B, Soto H, Ge N, Catron D, Buchanan ME, McClanahan T, Murphy E, Yuan W, Wagner SN, Barrera JL, Mohar A, Verastegui E, Zlotnik A (2001). Involvement of chemokine receptors in breast cancer metastasis. Nature.

[38] Zhang Z, Ni C, Chen W, Wu P, Wang Z, Yin J, Huang J, Qiu F (2014). Expression of CXCR4 and breast cancer prognosis: a systematic review and meta-analysis. BMC cancer.

[39] Yun J, Frankenberger CA, Kuo WL, Boelens MC, Eves EM, Cheng N, Liang H, Li WH, Ishwaran H, Minn AJ, Rosner MR (2011). Signalling pathway for RKIP and Let-7 regulates and predicts metastatic breast cancer. The EMBO journal.

[40] Eccles SA, Welch DR (2007). Metastasis: recent discoveries and novel treatment strategies. Lancet.

[41] Lu M, Lawrence DA, Marsters S, Acosta-Alvear D, Kimmig P, Mendez AS, Paton AW, Paton JC, Walter P, Ashkenazi A (2014). Cell death. Opposing unfolded-protein-response signals converge on death receptor 5 to control apoptosis. Science.

[42] Azijli K, Weyhenmeyer B, Peters GJ, de Jong S, Kruyt FA (2013). Non-canonical kinase signaling by the death ligand TRAIL in cancer cells: discord in the death receptor family. Cell death and differentiation.

[43] Trauzold A, Wermann H, Arlt A, Schutze S, Schafer H, Oestern S, Roder C, Ungefroren H, Lampe E, Heinrich M, Walczak H, Kalthoff H (2001). CD95 and TRAIL receptor-mediated activation of protein kinase C and NF-kappa B contributes to apoptosis resistance in ductal pancreatic adenocarcinoma cells. Oncogene.

[44] Buie HR, Campbell GM, Klinck RJ, MacNeil JA, Boyd SK (2007). Automatic segmentation of cortical and trabecular compartments based on a dual threshold technique for *in vivo* micro-CT bone analysis. Bone.

[45] Campbell GM, Tiwari S, Grundmann F, Purcz N, Schem C, Gluer CC (2014). Three-dimensional image registration improves the long-term precision of *in vivo* micro-computed tomographic measurements in anabolic and catabolic mouse models. Calcified tissue international.

[46] van de Wijngaert FP, Burger EH (1986). Demonstration of tartrate-resistant acid phosphatase in un-decalcified, glycolmethacrylate-embedded mouse bone: a possible marker for (pre)osteoclast identification. The journal of histochemistry and cytochemistry: official journal of the Histochemistry Society.

