# Supplementary material for: TRAIL-R2 promotes skeletal metastasis in a breast cancer xenograft mouse model
Source: Oncotarget. 2015 Mar 25;6(11):9502–16. doi: 10.18632/oncotarget.3321 (PMC4496234; doi:10.18632/oncotarget.3321)
Supplement: Supplementary file 1 [file oncotarget-06-9502-s001.pdf]

SUPPLEMENTARY FIGURE AND TABLES

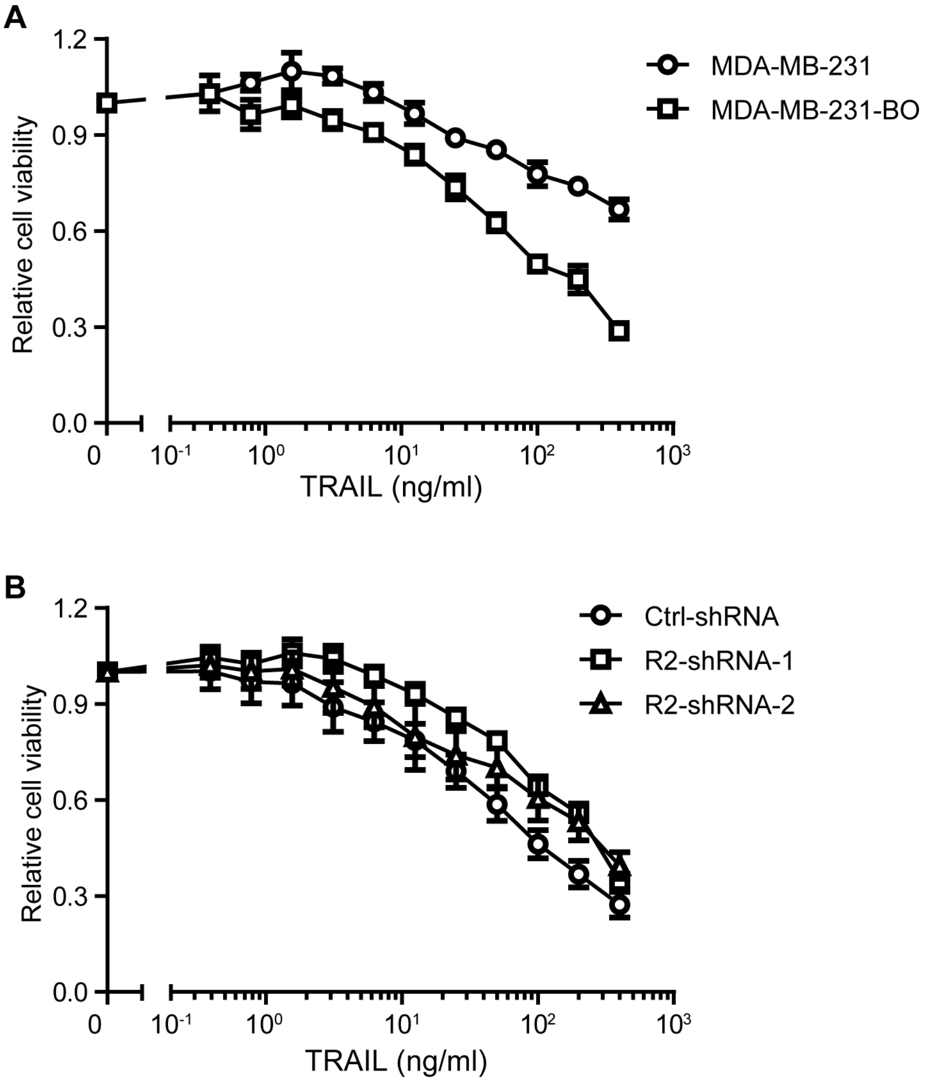

Supplementary Figure S1: Elevated levels of TRAIL-R2 are associated with greater sensitivity to TRAIL-mediated cell death. 24 h after seeding (96-well plates, 1 × 10^4 cells/well), parental MDA-MB-231 and the bone-homing MDA-MB-231-BO **(A)**, as well as control cells and cells expressing TRAIL-R2-shRNAs **(B)**, were treated for 48 h with increasing concentrations of TRAIL. Cell viability was assessed by crystal violet staining as previously described (Siegmund *et al*., (2005); Mol. Cell. Biol. 25, 6363). Graphs represent average values ± SD. (n = 6).

Supplementary Table S1: Quantification of FACS analyses performed on TRAIL-R2 knockdown cells TRAIL-R2 knockdown cells (R2-shRNA-1 or R2-shRNA-2) were analyzed in regard to the expression of TRAIL-R1, TRAIL-R2, CXCR4 and EGFR by flow cytometry. Percent of stained cells **(A)**, as well as staining intensities per cell **(B)**, relative to non-specific antibody controls, were quantified and fold changes relative to control calculated. Values represent fold change ± SD.

A	Non-permeabilized		Permeabilized	
	R2-shRNA-1	R2-shRNA-2	R2-shRNA-1	R2-shRNA-2
TRAIL-R2	0.54 ± 0.14	0.43 ± 0.10	0.24 ± 0.04	0.23 ± 0.06
TRAIL-R1	0.91 ± 0.28	1.00 ± 0.30	1.07 ± 0.10	1.06 ± 0.14
CXCR4	0.25 ± 0.09	0.24 ± 0.05	0.45 ± 0.04	0.40 ± 0.09
EGFR	1.01 ± 0.05	0.99 ± 0.05	1.00 ± 0.04	1.00 ± 0.04

B	Non-permeabilized		Permeabilized	
	R2-shRNA-1	R2-shRNA-2	R2-shRNA-1	R2-shRNA-2
TRAIL-R2	0.50 ± 0.15	0.53 ± 0.08	0.32 ± 0.08	0.32 ± 0.10
TRAIL-R1	0.95 ± 0.22	0.94 ± 0.22	0.94 ± 0.16	0.99 ± 0.15
CXCR4	0.36 ± 0.10	0.45 ± 0.10	0.30 ± 0.08	0.28 ± 0.08
EGFR	0.97 ± 0.09	0.98 ± 0.07	0.95 ± 0.09	0.94 ± 0.13

Supplementary Table S2: Quantification of FACS analyses performed on TRAIL-R2 overexpressing cells MDA-MB-231 cells overexpressing the long (R2-long) or short (R2-short) isoforms were analyzed by flow cytometry in regard to the expression of TRAIL-R2, CXCR4 and EGFR. Percent of stained cells **(A)**, as well as staining intensities per cell **(B)**, relative to non-specific antibody controls, were quantified and fold changes relative to control calculated. Values represent fold change ± SD.

A	Non-permeabilized		Permeabilized	
	R2-long	R2-short	R2-long	R2-short
TRAIL-R2	1.32 ± 0.24	1.62 ± 0.42	1.87 ± 0.24	1.99 ± 0.42
CXCR4	2.15 ± 0.14	2.39 ± 0.28	1.80 ± 0.24	1.79 ± 0.29
EGFR	0.99 ± 0.03	0.98 ± 0.04	0.99 ± 0.04	1.00 ± 0.05

B	Non-permeabilized		Permeabilized	
	R2-long	R2-short	R2-long	R2-short
TRAIL-R2	1.48 ± 0.26	1.67 ± 0.32	2.42 ± 0.28	2.33 ± 0.37
CXCR4	1.43 ± 0.25	1.49 ± 0.24	2.38 ± 0.44	2.37 ± 0.28
EGFR	0.99 ± 0.08	0.98 ± 0.06	0.98 ± 0.06	1.00 ± 0.07
